# Identification of novel *TCOF1* mutations in Treacher Collins syndrome and their functional characterization

**DOI:** 10.1186/s13023-025-03667-7

**Published:** 2025-04-16

**Authors:** Ying Chen, Run Yang, Xin Chen, Tianyu Zhang, Chenlong Li, Jing Ma

**Affiliations:** 1 Department of Facial Plastic and Reconstructive Surgery, ENT Institute, Eye and ENT Hospital of Fudan University, Shanghai, 200031 People’s Republic of China; 2 NHC Key Laboratory of Hearing Medicine, Fudan University, Shanghai, 200031 People’s Republic of China; 3 Institute of Medical Genetics and Genomics, Fudan University, Shanghai, 200032 People’s Republic of China; 4 Surgery Laboratory, Institute of Medical Sciences, General Hospital of Ningxia Medical University, Yinchuan, 750004 Ningxia People’s Republic of China

**Keywords:** Treacher Collins syndrome, *TCOF1*, Mutation, Pathogenesis

## Abstract

**Background:**

Treacher Collins syndrome (TCS) is a congenital disorder primarily caused by the mutation in the Treacle Ribosome Biogenesis Factor 1 (*TCOF1*) gene. However, the significance of many *TCOF1* mutations remains uncertain.

**Results:**

We report two novel mutations identified in two TCS families and assess their pathogenicity alongside two previously reported mutations. Both novel mutations, c.2115dupG (p.T706DfsTer52) and c.2142+23_2142+52 del (p.A715VfsTer31), result in truncated proteins lacking nuclear location signals (NLSs), which impedes their entry into the nucleus and reduces mRNA expression level. Notably, the mutation c.2142+23_2142+52 del, leading to the retention of a 62 bp intron and disrupting RNA splicing, represents the first documented case of intron retention in TCS patients. Additionally, the previously reported mutation c.136 C> G (p.L46V) hinders protein nuclear location, while mutation c.1719del (p.N574TfsTer22) significantly decreases mRNA levels.

**Conclusions:**

Our research expands the spectrum of *TCOF1* mutations and provides evidence clarifying their pathogenic nature. These findings are crucial for genetic counseling and prenatal diagnosis for TCS patients.

**Supplementary Information:**

The online version contains supplementary material available at 10.1186/s13023-025-03667-7.

## Background

Treacher Collins syndrome is a monogenic inherited disease due to the abnormal development of the first and second pharyngeal arches, affecting approximately 1 in 50,000 live births [1]. TCS falls within the spectrum of mandibulofacial dysostosis (MFD) and is distinguished by facial features often described as a ‘fish-like’ appearance [2]. Common manifestations include downslanting palpebral fissures, bilateral malar and mandibular hypoplasia, microtia, eyelids coloboma, cleft palate, and posterior nasal choanal atresia [3]. Hearing loss is common, affecting about 83%–92% of individuals with TCS [3]. Additionally, a minority of cases may present with extracraniofacial anomalies, including congenital heart defects, renal abnormalities, and anomalies of the limbs or thumbs [4]. These anomalies present significant medical challenges requiring multidisciplinary care and have profound psychosocial impacts on affected individuals and their families. The severity of Treacher Collins syndrome phenotypes varies significantly, both within families and between different families. Mild cases may have subtle symptoms that could lead to underdiagnosis, while severe cases may experience feeding or respiratory difficulties, and in extreme cases, neonatal mortality [5]. Moreover, the craniofacial phenotypes associated with TCS often overlap with those of other MFD syndromes, such as CHARGE syndrome, mandibulofacial dysostosis with microcephaly, Nager syndrome or oculo-auriculo-vertebral spectrum [6], complicating the accurate diagnosis of this condition. As a result, genetic testing plays a crucial role in the definitive diagnosis of TCS.

The genetic basis of TCS primarily involves heterozygous pathogenic mutations in *TCOF1* (OMIM* 606847), which account for approximately 63%–93% of TCS cases [7, 8]. Additional genes implicated in TCS include *POLR1D* (OMIM* 613715), *POLR1C* (OMIM* 610060) and *POLR1B* (OMIM* 618939), which together are responsible for 11% to 23% of cases in individuals without *TCOF1* mutations. Unfortunately, in 4%–10% of TCS cases, pathogenic mutations remained undetected, even among those displaying typical phenotypes [7]. Approximately 60% of TCS cases follow an autosomal dominant inheritance pattern involving *TCOF1*, *POLR1D*, and *POLR1B*, or an autosomal recessive inheritance pattern involving *POLR1D* and *POLR1C*, while the remainders are de novo cases. Identifying the genetic basis of TCS is vital for distinguishing it from other MFD syndromes and informing genetic counseling and prenatal diagnosis. This knowledge significantly contributes to the prevention of births affected by TCS.

The *TCOF1* gene, located on the long arm of chromosome 5, encodes the treacle protein, which regulates ribosome biogenesis during embryogenesis by interacting with upstream binding factor (UBF) [9]. Studies utilizing *Tcof1*^*+/−*^ knockout mouse models have underscored treacle’s vital role in craniofacial development [10]. Haploinsufficiency of treacle disrupts mature ribosome production in neuroepithelial cells and neural crest cells (NCCs), which are crucial for the development of craniofacial bones, cartilage, and soft tissues [11]. Additionally, treacle plays a significant role in antioxidative defense, particularly in protecting cells from DNA damage by regulating rRNA transcription [12]. Mutations in *TCOF1* affecting ribosome biogenesis can lead to decreased proliferation in neuroepithelial cells, reduced numbers of migratory cranial NCCs, and cranio-skeletal hypoplasia, all contributing to the clinical manifestations of TCS [10, 13]. To date, over 300 *TCOF1* mutations associated with TCS have been documented in The Human Gene Mutation Database (https://www.hgmd.cf.ac.uk/). Despite this extensive cataloging, the mutation spectrum is gradually decreasing. Nonetheless, the uncertain pathogenesis of certain detected mutations poses challenges in offering accurate genetic diagnosis for TCS patients, complicating the provision of genetic counseling and prenatal diagnosis.

In this study, we discovered a novel duplication and a novel intron deletion mutation in the *TCOF1* gene in two TCS families using whole-exome sequencing (WES). Subsequently, we assessed the pathogenicity of these novel mutations, in addition to two previously reported mutations, using in vitro experiments. Our findings not only contribute new mutation information for enhancing TCS diagnosis and genetic counselling but also furnish functional evidence elucidating the pathogenicity of these mutations.

## Methods

### Clinical samples

All participants were recruited from the Eye & ENT hospital of Fudan University. Peripheral blood samples were obtained for DNA extraction using BD Vacutainer® EDTA Tubes (Becton Dickinson, NJ, USA) and for RNA extraction using PAXgene® Blood RNA Tube (QIAGEN, Hombrechtikon, Switzerland).The collected peripheral blood samples were stored at −20℃. All procedures were carried out with the approval of the Ethics Committees of Eye & ENT hospital of Fudan University (2020069). Written informed consent for participation in this study was obtained from all participants or their legal guardians.

### Whole exome sequencing and mutational analysis

Genomic DNA was extracted from peripheral blood using the DNeasy 96 Blood & Tissue Kit (QIAGEN, Hombrechtikon, Switzerland) for WES. DNA libraries prepared from 0.2 μg of qualified genomic DNA underwent purification by the Ampure XP system (Beckman Coulter, Beverly, USA), and were quantified with the Qubit BR ssDNA kit (Agilent Technologies, Waldbronn, Germany). Paired-end 150 bp sequencing was performed on the DNBSEQ-T7 platform at the Medical Laboratory of Nantong ZhongKe Co, Ltd (Jiangsu, China). Sequencing data were collected, filtered and aligned to the human reference genome assembly GRCh37/hg19 using the Burrows-Wheeler Aligner [14] and Binary Alignment Map (BAM) files were generated by the SAMtools. Single-nucleotide polymorphisms (SNPs) and small insertions and deletions (InDels) for each exome were detected using Genome Analysis Toolkit software [15] and Cnvkit [16]. Variations were annotated by ANNOVAR [17] and SnpEff [18], and those that were synonymous, in segment duplication, or had a minor allele frequency (MAF) > 0.01 were excluded. Detected variations were characterized and prioritized using public databases such as dbSNP (https://www.ncbi.nlm.nih.gov/SNP/), 1000 Genome, the Consensus Coding Sequence (CCDS), RefSeq, Ensembl, and UCSC Genome Browser. The nomenclature for *TCOF1* variations followed Human Genome Variation Society (HGVS) guidelines (http://www.hgvs.org) using the reference sequence NM_001135243.1.

Following identification through WES, the *TCOF1* mutations underwent validation and segregation analysis within the respective families. Peripheral blood DNA extracted from the probands and their family members was subjected to PCR amplification, followed by Sanger sequencing on a 3730xl DNA Analyzer (Applied Biosystems, Warrington, UK). Sequencing results were analyzed by Chromas 2.6.6 (Technelysium Pty Ltd, South Brisbane, Australia).

We employed SIFT, PolyPhen-2, and CADD analyses to predict the pathogenicity of missense variants, assessing their potential impact on protein function. Additionally, we utilized Human Splicing Finder (http://www.umd.be/HSF) to predict the splicing effects of identified mutations, and elucidate potential alterations in mRNA splicing patterns. Subsequently, SWISS-MODEL was employed to predict the three-dimensional (3D) structures of both wild-type and mutant-type proteins, providing insights into structural changes that may occur due to the identified mutations.

### RNA extraction, reverse transcription PCR and quantitative real-time PCR analysis

RNA extraction was performed using the PAXgene™ Blood RNA System Kit (QIAGEN, Hombrechtikon, Switzerland) following the manufacturer’s guidelines. The concentration and purity of RNA extracts were determined using the NanoDrop 2000 spectrophotometer (Labtech International, Ringmer, UK), and RNA integrity was assessed through electrophoresis. For reverse transcription PCR (RT-PCR) and quantitative real-time PCR (qPCR), RNA was retro-transcribed using the Hifair™ III 1st strand cDNA Synthesis SuperMix (Yeasen, Shanghai, China) according to the manufacturer’s instructions. Subsequently, PCR amplification of *TCOF1* was performed on each sample in a 50 µL reaction using 100 ng of cDNA by the Premix Taq™ (LA Taq™ Version 2.0 plus dye) (Takara bio, Kusatsu, Japan). The PCR primer pair designed to encompass *TCOF1* exons 12 through 14, which include the region with the splicing variant, is listed in Table S1. The PCR reaction conditions were as follows: 98 °C for 3 min, followed by 40 cycles of 98 °C for 15 s, 60 °C for 15 s, and 72 °C for 30 s, and a final extension at 72 °C for 5 min. The amplification product was verified using a 1.0% agarose gel (Sigma-Aldrich, MO, USA) before Sanger sequencing. Sequences analysis was carried out by Chromas (version 1.62, Technelysium Pty Ltd). To confirm the mRNA expression levels of *TCOF1* and 45S rRNA, qPCR was performed using the TB Green® Fast qPCR Mix (Takara, Osaka, Japan) and the cDNA synthesized in the previous step on the StepOnePlus™ Real-Time PCR System (ThermoFisher Scientific, CA, USA). The cycling condition were: 30 s at 95 °C, and 40 cycles of 5 s at 95 °C and 30 s at 60 °C. The 2^−ΔΔCT^ method were applied to calculate the relative *TCOF1* and 45S rRNA expression level normalized to *ACTB*. The primer pairs for qPCR are listed in Table S1.

### Cell culture

The HEK293T cell line (SB-C01005) was obtained from share-bio (Shanghai, China) and cultured in Dulbecco’s Modified Eagle Medium (DMEM) (Biological Industries, Beit HaEmek, Israel) supplemented with 10% Fetal Bovine Serum (FBS) (Gibco Laboratories, CA, USA) and 1% penicillin–streptomycin (NCM Biotech, Suzhou, China). Cultures were maintained in a humidified incubator at 37 °C with 5% CO_2_.

### Plasmid construction and transfection

The entire coding sequence of *TCOF1* was cloned into the pCDH-CMV-MCS-EF1-Puro eukaryotic vector (GeneRay, Shanghai, China). In addition, a DYKDDDDK peptide sequence (FLAG) was added to the N-terminus of TCOF1 protein. Mutants of TCOF1 were generated using the Q5 Site-Directed Mutagenesis Kit (E0554, New England BioLabs, Beverly, MA, USA) and verified by Sanger sequencing. HEK293T cells were cultured in 24 well dishes (In Vitro Scientific, CA, USA) until reaching 80% confluence. Then, the wild-type and three mutant FLAG-TCOF1 constructs were transfected into HEK293T cells using the Lipofectamine™ 3000 Transfection Reagent (Thermo Fisher, Paisley, UK) according to the transfection protocol.

### Immunofluorescence staining and confocal microscopy imaging

At 96 h post-transfection, cells were subjected to immunofluorescence (IF) staining using the primary antibody DYKDDDDK-Tag(3B9) mAb (Abmart, Shanghai, China) at a 1:200 dilution. Subsequently, cells were stained with the secondary antibody Alexa Fluor® 488 AffiniPure Goat Anti-Mouse IgG (H + L) (Yeasen, Shanghai, China) at a 1:200 dilution, followed by staining with DAPI stain solution at a concentration of 10 μg/mL. Imaging was performed using the Leica TCS SP8 MP Multiphoton Microscope (Leica, Germany). Image analysis was conducted using ImageJ (version 1.53, National Institutes of Health, USA).

### Western blot assay

At 48 h post-transfection, HEK293T cells were harvested and lysed using Pierce® RIPA buffer (Thermo Scientific, MA, USA). Protein concentration was determined using the BCA Protein Assay Kit (Beyotime Biotechnology, Shanghai, China). Subsequently, proteins were separated using a 15% Tris-Gly gel (Epizyme, Shanghai, China) and transferred onto an Immobilon-P PVDF membrane (Millipore, MA, USA). The membranes were then blocked with 5% skimmed milk for 90 min and incubated overnight at 4℃ with primary antibodies. Afterward, tthey were incubated with HRP-conjugated secondary antibodies for 1 h at room temperature. The blots were developed using Chemiluminescent HRP Substrate reagent (Millipore MA, USA). The FLAG-tag was detected by the primary antibody DYKDDDDK-Tag (3B9) mouse mAb (Abmart, Shanghai, China) at a 1:1,000 dilution, with HRP-conjugated Affinipure Goat anti-mouse lgG(H + L) (SA00001-1, Proteintech, IL, USA) as the secondary antibody at a 1:1,000 dilution. The housekeeping protein GAPDH was used as a control, detected using primary antibody GAPDH Rabbit mAb (14C10, Cell Signaling Technology, MA, USA) at a 1:1,000 dilution, and the secondary antibody HRP-conjugated Affinipure Goat Anti-Rabbit IgG(H + L) (SA00001-2, Proteintech, IL, USA) at a 1:2,000 dilution.

### Statistical analysis

All statistical analyses were performed by paired two-tailed Student’s t-test with GraphPad Software (La Jolla, CA, USA). A *P* value < 0.05 was considered statistically significant.

## Results

### Patients exhibit typical features of Treacher Collins syndrome

**Fig. 1:**
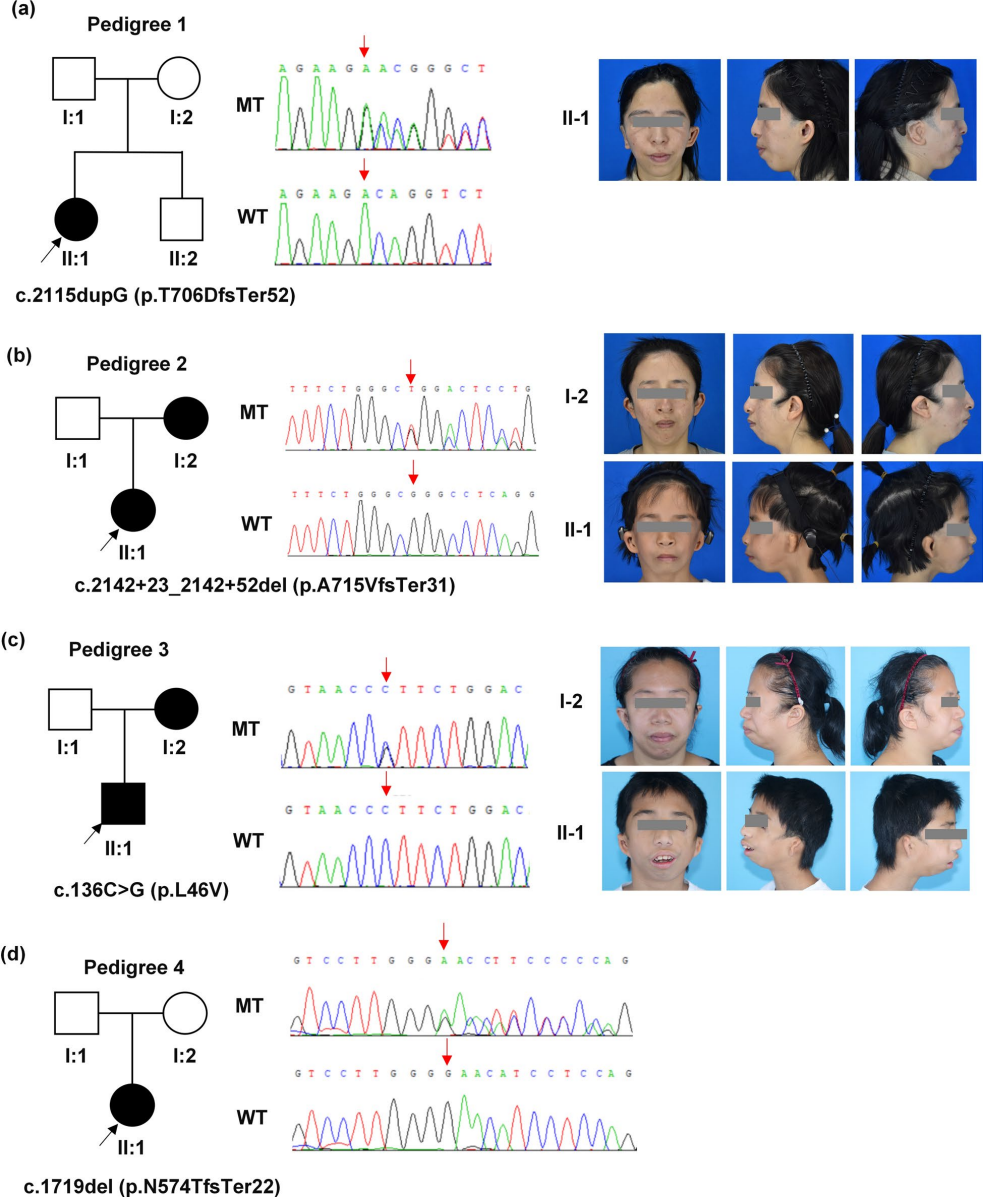
Four Chinese families with TCS-associated *TCOF1* mutations. **a**-**d** The pedigrees, genotypes, Sanger sequencing electropherograms, and phenotypes of four unrelated families. In the pedigrees, solid symbols represent affected individuals, whereas open symbols denote unaffected family members. Circles and squares indicate females and males, respectively. Black arrows indicate the probands. Pedigree 4 has not granted permission for photo usage. WT: wild-type; MT: mutant

The study recruited a total of four unrelated nuclear families with TCS, as illustrated in Fig. 1. These families consisted of six individuals affected by TCS and their healthy family members. Notably, two of the families (Pedigree 3 and Pedigree 4) had been previously reported [19, 20] and were referred to the clinic for hearing rehabilitation surgeries. They were included in this study because the affected individuals had not been thoroughly documented, evaluations had not been conducted on their family members, and the pathogenic mechanisms of the *TCOF1* mutations present in these patients remained unclear.

During clinical examination, all affected individuals displayed characteristic phenotypes of TCS, including down-slanting palpebral fissures, lower eyelid coloboma, mandibular hypoplasia, malocclusion, bilateral ear deformities and hearing loss. Notably, none of the patients showed indications of intellectual disability or mental retardation. Further assessments uncovered additional phenotypes, such as facial cleft in patient II:1 from Pedigree 3 and strabismus in patient II:1 from Pedigree 4. A summary of the observed phenotypes in the TCS patients is presents in Table 1.

**Table 1:** Clinical manifestations and identified mutations

Pedigree		1	2		3		4
Patient ID		II:1	II:1	I:2	II:1	I:2	II:1
Sex		F	F	F	M	F	F
Age on examination		32	14	37	11	36	9
DNA change		c.2115dupG	c.2142+23_2142+52 del		c.136C>G		c.1719del
Protein change		p.T706DfsTer52	p.A715VfsTer31		p.L46V		p.N574TfsTer22
Location		Exon 12	Intron 12		Exon 2		Exon 11
Mutation type		Frameshift	Splicing		Missense		Frameshift
SIFT/Polyphen- 2/CADD		/	/		D/PD/P		/
ACMG classification		PVS1+PM2_Supporting+PP4	PM2_Supporting+PP4		PM2_Supporting+PP4		PVS1+PM2_Supporting+PM6+PP4
Reference		Novel	Novel		Chen et al. [19]		Zhang et al.[20]
Medical history	Family history	–	+	+	+	+	–
	Intubation or tracheostomy	–	–	–	–	–	–
	Nasogastric tube or gastrostomy	–	–	–	–	–	–
	Plastic surgery	+	+	–	+	–	–
	Hearing rehablitation or cholesteatoma removal	+	+	–	–	+	–
Face	Mandibular hypoplasia	+	+	+	+	+	+
	Malar hypoplasia	+	+	+	+	+	+
	Asymmetry	–	–	+	+	–	+
Eye	Downward-slanting palpebral fissures	+	+	+	+	+	+
	Coloboma of the lower lid	+	+	+	+	+	+
	Strabismus	–	–	–	–	–	+
Ear	Hearing loss	+	+	+	+	+	+
	Atresia/stenosis of EAC	+	+	–	+	–	+
	auricle malformation	+	+	+	+	+	+
Other	Preauricular hair displacement	+	+	+	+	+	–
	Cleft palate	–	–	–	–	–	–
	Macrostomia	–	–	–	–	–	+
	Choanal stenosis/atresia	–	–	–	–	–	–
Development	Short stature	–	+	+	–	–	–
	Delayed speech development	–	–	–	+	–	–

*D* damaging; *PD* probably damaging; *P* pathogenic; *EAC* external auditory canal

### Genetic finding through whole exome sequencing and Sanger sequencing validation

**Fig. 2:**
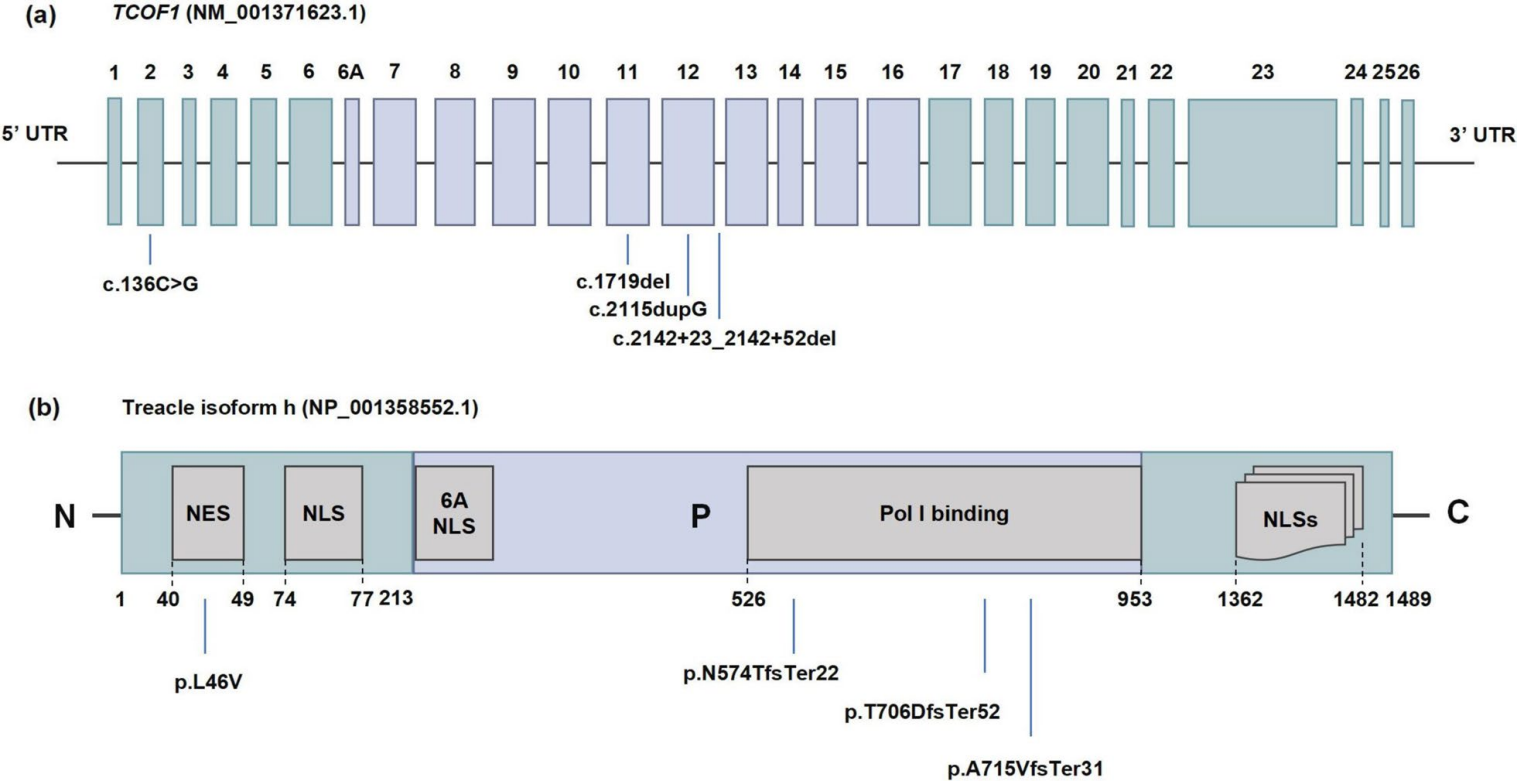
The mapping of *TCOF1* mutations. **a** Schematic representation of *TCOF1* transcript variant 8 (NM_001371623.1), highlighting the locations of four mutations. **b** The structure of the treacle isoform h (NP_001358552.1) encoded by *TCOF1* transcript variant 8. This isoform comprises 27 exons, including exon 1–26 and an additional exon 6A. The N-terminus region (N), encompassing exons 1–6 (amino acids 1–213), contains both a nuclear export signal (NES, amino acids 40–49) and a nuclear localization signal (NLS, amino acids 74–77). The central phosphorylated domain (P) spanning from exons 6A to exon 16 (amino acids 214–953), containing a motif responsible for binding to RNA polymerase I (Pol I binding) (amino acid 526–953). The C-terminus region (C) from exon 17 to exon 26 features multiple NLSs within exons 23, 24 and 25

Through WES and subsequent Sanger sequencing validation, we discovered a novel *TCOF1* heterozygous mutation c.2115dupG p.T706DfsTer52 in Patient II:1 from Pedigree 1. This mutation was absent in her unaffected parents and brother (Fig. 1a). In Pedigree 2, both Patient II:1 and her affected mother I:2 were found to carry a novel *TCOF1* heterozygous splicing site mutation c.2142+23_2142+52del p.A715VfsTer31, while the wild-type *TCOF1* alleles were present in her unaffected father (Fig. 1b). To our knowledge, these two mutations have not been reported in Human Gene Mutation Database or other existing literatures. Furthermore, no pathogenic mutations were detected in other genes associated with TCS. Two previously reported mutations, namely c.136C>G in Pedigree 3 and c.1719del in Pedigree 4, were also verified through Sanger Sequencing (Fig. 1c and d). A summary of all identified mutations is provided in Table 1 and Fig. 2.

### Functional consequences of* TCOF1* mutation c.2115 dupG

**Fig. 3:**
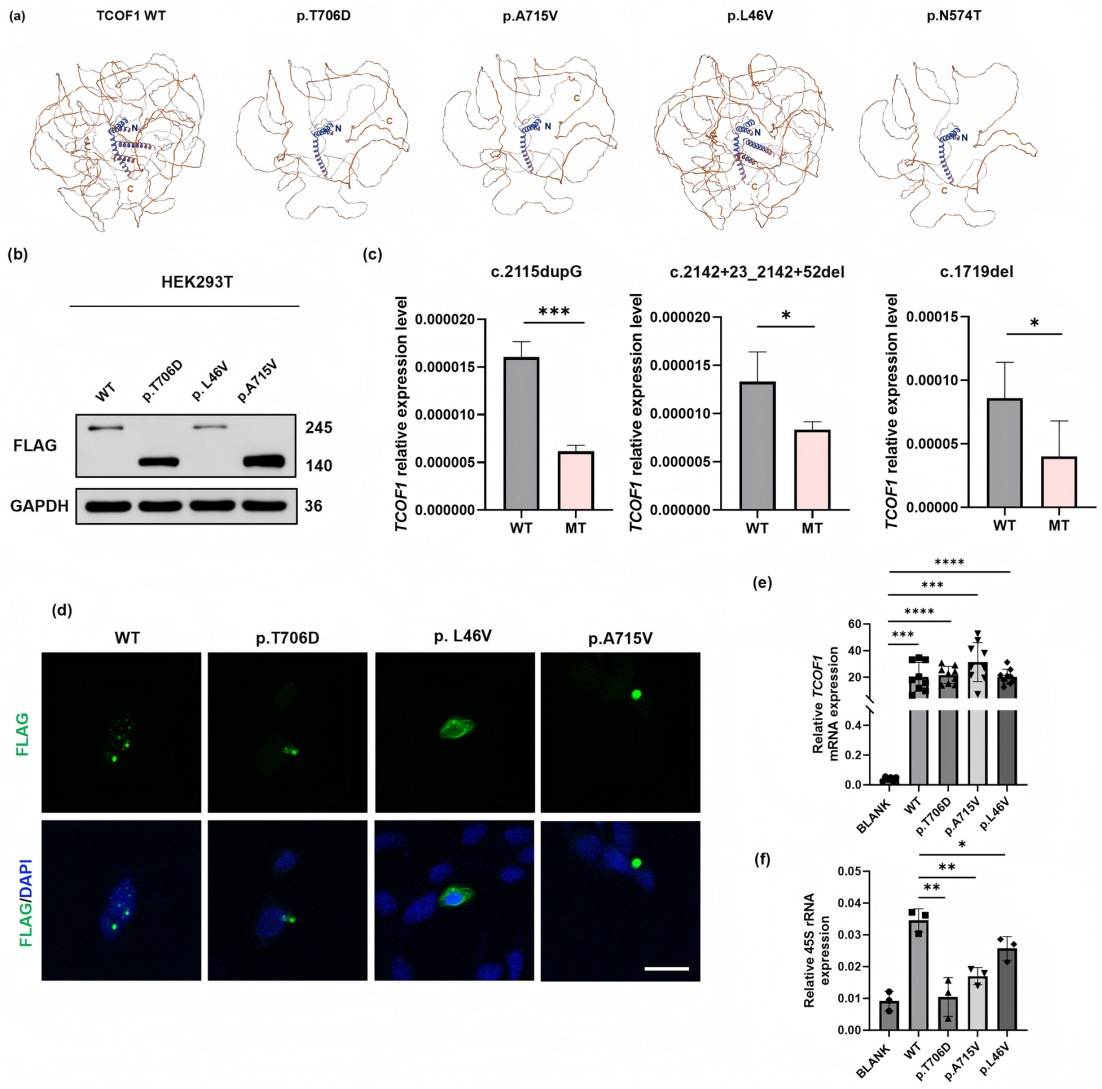
Mutations in four Chinese families and their pathogenic effects. **a** 3D modeling of mutant proteins using SWISS-MODEL to illustrate structural alterations. **b** Anti-FLAG western blot assay demonstrating truncation of mutant proteins p.T706DfsTer52 and p.A715VfsTer31 at approximately 140 kDa, whereas the mutant protein p.L46V exhibits a size similar to the wild-type at 245 kDa. **c** *TCOF1* mRNA expression in patient (MT) and healthy family member as control (WT). **d** Subcellular distribution of treacle indicated by anti-FLAG immunofluorescence. Scale bar = 100 μm. Each experiment was repeated at least three times. **e** *TCOF1* mRNA overexpression validated by qPCR in HEK293T cells. **f** The relative expression of 45S rRNA validated by qPCR in HEK293T cells. *P* values obtained from two-tailed Student’s t test. *n* = 3 biologically independent samples. Error bars represent standard errors. Significance levels are indicated as *, *P* < 0.05; ***, *P* < 0.001

The c.2115dupG mutation in *TCOF1* leads to a premature stop codon, resulting in a truncated protein that is 732 amino acids shorter than its wild-type counterpart. To investigate the pathogenicity of this mutation, we transfected FLAG-tagged *TCOF1* into HEK293T cell lines to examine its effect at the protein level. Western blotting (WB) with an anti-FLAG antibody revealed that the mutant protein displayed as a band around 140 kDa, contrasting with the 245 kDa band observed for the wild-type protein (Fig. 3b), confirming the truncation of the protein. Notably, the truncated protein lacked the RNA pol I binding domain and C-terminus, which contains several nuclear localization signals (NLSs) (Fig. 2b). To further investigate the subcellular localization of Treacle, we employed IF staining using the anti-FLAG antibody. The results revealed that wild-type Treacle predominantly localized to the nucleus of HEK293T cells, with minimal cytoplasmic expression. In contrast, the mutant protein was primarily detected in the cytoplasm, with limited presence in the nucleus (Fig. 3d).

Given that a premature stop codon can trigger mRNA degradation through the Nonsense-Mediated Decay (NMD) mechanism, we conducted qPCR to investigate *TCOF1* mRNA expression in Pedigree 1. The results showed a significant decrease in *TCOF1* mRNA expression levels in the patient compared to her unaffected parent, who served as the normal control (*P* < 0.001) (Fig. 3c).

In summary, the *TCOF1* c.2115dupG mutation creates a truncated protein missing NLSs, leading to decreased entry into the nucleus and mRNA degradation.

### Functional impact of *TCOF1* mutation c.2142+23_2142+52del

**Fig. 4:**
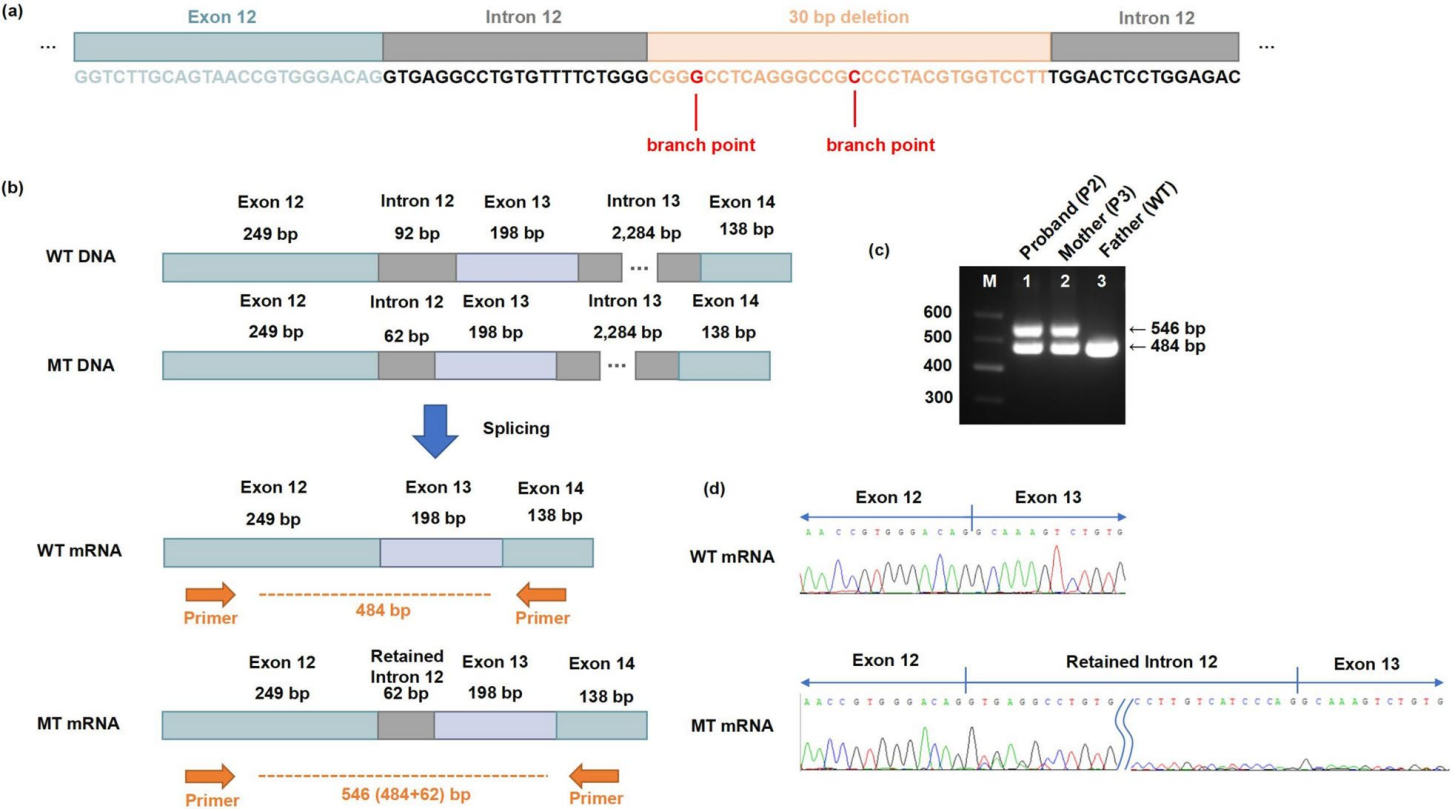
The disruption of mRNA splicing by mutation c.2142+23_2142+52del **a** Human Splicing Finder prediction suggesting the loss of two predicted branch points within the 30 bp deletion (orange box). **b** Schematic representation of the mutational effects on splicing. The wild-type (WT) mature mRNA features the exon 12 followed by exon 13 and exon 14. In contrast, the mutant-type (MT) mature mRNA contains a 62 bp retained intron sequence between exon 12 and exon 13, as denoted by the gray box. **c** PCR products confirming the altered splicing visualized on a 1.0% agarose gel. Lane M: DNA ladder. The WT PCR product is 484 bp in length, where the MT PCR product displays an additional band of 546 bp. **d** Sanger sequencing electropherograms of the RT-PCR products, showing the 484 bp WT band (upper panel) and the 546 bp MT band (lower panel)

Using the Human Splicing Finder (http://www.umd.be/HSF/), the c.2142+23_2142+52del mutation in Pedigree 2 was predicted to result in the loss of two potential branch points with intron 12, consequently impeding mRNA splicing (Fig. 4a). RT-PCR analysis of *TCOF1* exon 12–14 in the proband and her affected mother revealed an additional band of approximately 546 bp, in contrast to the single band of approximately 484 bp observed in the unaffected father (Fig. 4c). Sanger sequencing confirmed that the 484 bp band represented the wild-type transcript, while the 546 bp band contained a partially retained 62 bp of intron 12 (Fig. 4d), confirming the loss of branch points and disruption of the splicing process between exon 12 and exon 13 (Fig. 4c).

The insertion of the 62 bp intron into *TCOF1* mRNA caused a frameshift mutation and an introduction of a premature stop codon. This, in turn, resulted in protein truncation and the loss of the NLSs (Fig. 2b). To further investigate the effects of c.2142+23_2142+52 del on protein expression and subcellular localization, FLAG-tagged WT and mutant *TCOF1* plasmids were constructed. In HEK293T cells transfected with the c.2142+23_2142+52del mutation, an aberrant band at approximately 140 kDa was detected by WB assay (Fig. 3b). IF analysis revealed that the mutant Treacle protein p.A715VfsTer31 accumulated outside the nucleus, whereas the wild-type protein primarily distributed within the nucleus (Fig. 3d). Moreover, qPCR results indicated a significant reduction in *TCOF1* mRNA expression in the affected individual from Pedigree 2 when compared to her healthy father (Fig. 3c).

In short, the *TCOF1* mutation c.2142+23_2142+52 del also results in protein truncation through abnormal splicing, thereby affecting the entering of treacle into the nucleus and leading to mRNA degradation.

### Disruption of subcellular distribution by* TCOF1* mutation c.136 C > G

The c.136 C > G missense mutation in *TCOF1* is classified as pathogenic, attributed to its high conservation within the *TCOF1* sequence, its low frequency in the healthy population, and its co-segregation within Pedigree 3 [19]. Despite being recognized as pathogenic, the exact mechanism of its pathogenicity remains unclear. This mutation induces an amino acid substitution at the N-terminus of Treacle (Fig. 2). Nevertheless, the substitution does not alter the hydrophobicity of the amino acid, nor does it induce significant changes in the protein conformation (Fig. 3a). In HEK293T cells transfected with *TCOF1* c.136C>G plasmids, WB analysis result showed that the mutant protein (p.L46V) appeared at approximately 245 kDa, indicating that the size of the mutant protein closely resembles that of the WT treacle (Fig. 3b). However, an IF assay demonstrated that the mutant protein is dispersed throughout both the nucleus and the cytoplasm. In contrast, the WT protein was rarely detected in the cytoplasm (Fig. 3d).

Therefore, despite the similar size, the altered subcellular distribution of the mutant protein suggests a functional impact associated with the c.136C>G mutation in *TCOF1*.

### Impact of *TCOF1* mutation c.1719del on mRNA expression

The c.1719 del mutation, located in exon 11, was initially identified by Zhang et al. [20]. This mutation is predicted to introduce a premature termination codon, leading to the production of a truncated protein without NLSs at the C-terminus (Fig. 2b). The truncated treacle protein produced by *TCOF1* c.1719del mutation is expected to be shorter than that generated by the *TCOF1* c.2115dupG mutation, leading to a loss of more functional domains. Consequently, we postulated that the c.1719del should share a similar pathogenic mechanism with c.2115dupG. So, we only conducted a qPCR assay using peripheral blood RNA from the patient and her healthy parents. The results revealed a significant reduction in *TCOF1* mRNA expression level in the patient, compared to her healthy father (Fig. 3c).

This observation suggests that the c.1719del mutation leads to a decreased expression of the mRNA, reinforcing its potential pathogenicity and underscoring similarities with the c.2115dupG mutation in terms of molecular consequences.

### Mutations of *TCOF1* affect ribosome biogenesis

To investigate whether the rare *TCOF1* variants affect ribosome biogenesis, we extracted total RNA from transfected HEK293T cells, with transfection efficiency shown in Fig. 3e. Following the method of Wang et al. (2023) [37], we assessed the expression levels of 45S pre-rRNA to evaluate the impact of *TCOF1* mutations on ribosome biogenesis. Consistent with previous studies, the p.T706D, p.L46V, and p.A715V variants led to a significant reduction in 45S pre-rRNA mRNA levels (Fig. 3f).

## Discussion

To date, more than 300 mutations in *TCOF1* have been reported, however, the pathogenic variants responsible for approximately 11% of TCS cases remain unidentified [21]. Among all identified *TCOF1* mutations, deletions represent the most prevalent pathogenic mutations (59.6%), followed by substitutions (29.8%) and insertions (10.7%) [22]. Splicing mutations, which account for about 7%−16% of affected TCS individuals [23], have been reported to cause the inactivation of splice sites [24, 25], intron retention [24] and/or exon skipping [26, 27]. In our study, we identified a novel intronic splicing mutation in *TCOF1*, c.2142+23_2142+52del, which results in the loss of two potential branch points and causes 62 bp retention of intron 12. This mutation disrupts normal mRNA splicing, resulting in the introduction of a premature stop codon. Interestingly, similar to findings in previous studies [24], we observed a premature termination codon is created, which causes a truncated protein. Furthermore, qPCR analysis confirmed a reduction in transcript levels of the mutant allele compared to the wild-type allele. This is consistent with previous studies reporting reduced transcription levels of *TCOF1* by 18–31% in TCS patients [28]. This reduction is attributed to the activation of the NMD triggered by premature stop codons.

Moreover, the two novel mutations identified in this study, c.2115dupG and c.2142+23_2142+52del, are located in exons that are less commonly associated with mutations, yet still within the central repeat domain (CRD) [21]. This finding expands the known mutational spectrum of *TCOF1*. Although these mutations do not coincide with the frequently reported hotspots in exons 8, 10, and 23, which are prevalent in both Caucasian and Asian populations [19, 21], they are still within a critical functional region of the gene.

Over the past five years, significant progress has been made in understanding the functional implications of *TCOF1* gene mutations. The treacle protein encoded by *TCOF1* contains key regulatory signals, including nuclear localization signals (NLSs) and a nuclear export signal (NES) [29]. These signals facilitate treacle’s dynamic shuttling between the nucleus and cytoplasm, ensuring its proper function in rRNA transcription [30, 31]. The NLSs, primarily located in the N-terminus and C-terminus, are crucial for nuclear import, while the NES at the N-terminus regulates its export [30, 32]. Disruptions in these signals can impair treacle’s nuclear localization, affecting its role in transcription and contributing to TCS pathogenesis [32]. Our study introduces a novel perspective by identifying mutations located at NES. While earlier studies have identified the location of the NES, they have not investigated its functional role on treacle’s location [29]. Through in vitro experiments, we demonstrate that a missense mutation (c.136C>G), located in the NES and near the NLS within the N-terminus, caused decrease in treacle’s presence in the nucleus. This effect may arise from the substitution disrupting the interplay between the NLS and the NES [33]. Consequently, these mutations disrupt the transcriptional regulation of treacle in the nucleus, impairing its function, and thereby contributing to pathogenicity associated with TCS.

Ribosome biogenesis is a process essential for normal craniofacial development and begins in the nucleolus, where treacle is actively involved in rDNA transcription, pre-rRNA processing, and ribosomal assembly [34, 35]. In mammals, this process starts with the transcription of 45S pre-rRNA from rDNA [36, 37]. Similar to previous stuidies [37], our results shows that overexpression of mutated *TCOF1* significantly reduces 45S rRNA mRNA levels, suggesting that these mutations may mislocalize treacle, thereby disrupting ribosome biogenesis. Given the fundamental role of ribosome biogenesis in craniofacial development [35], such impairments may be a key contributor to TCS pathogenesis.

Our research employs routine in vitro experimental methods to investigate the pathogenicity of *TCOF1* mutations. These methods are simple, cost-effective, and time-efficient, making them well-suited for clinical laboratory testing. The platform we developed provides more reliable evidence for gene pathogenicity predictions, enhancing the accuracy of genetic diagnoses for TCS patients. In addition, the validation of pathogenicity for other disease genes can also be facilitated. Appropriate technical approaches can be selected based on the structure and function of the proteins encoded by the gene. However, we acknowledge that our small sample size and limited range of *TCOF1 *mutations may impact the generalizability of our findings. Future research with larger and more diverse cohorts is needed to further validate our results.

## Conclusions

In summary, this study contributes valuable gene mutation information for the genetic diagnosis of TCS and establishes a practical experimental framework for assessing gene pathogenicity. These advancements are crucial for guiding future genetic counseling or prenatal diagnosis efforts.

## Supplementary Information

Supplementary material 1.

## Data Availability

The data and materials that support the findings of this study are openly available in Genome Sequence Archive for Human at https://ngdc.cncb.ac.cn/search/specific?db=hra&q=HRA006969, reference number HRA006969.

## Abbreviations

IF: Immunofluorescence
MFD: Mandibulofacial dysostosis
NLS: Nuclear localization signal
NMD: Nonsense-mediated mRNA decay
qPCR: Quantitative real-time PCR
WES: Whole-exome sequencing
WB: Western blot
