# Supplementary material for: Identification of novel TCOF1 mutations in Treacher Collins syndrome and their functional characterization
Source: Orphanet J Rare Dis. 2025 Apr 16;20:184. doi: 10.1186/s13023-025-03667-7 (PMC12001626; doi:10.1186/s13023-025-03667-7)
Supplement: Supplementary file 1 — Supplementary material 1. [file 13023_2025_3667_MOESM1_ESM.docx]

**Supplentary Information**

**Table S1. Primers used in the present study**

| Assays | Gene | Primer | Nucleotide sequence |
| --- | --- | --- | --- |
| Real-time PCR | *TCOF1* | Forward (exon 13) | 5′- TACCACTGCATCTGCCAAGG -3′ |
|  |  | Reverse (exon 15) | 5′- TCCAGGGGCTGAAATTGTCC -3′ |
| Quantitative real-time PCR | *TCOF1* | Forward | 5′- CGGGAGCTACTTCCCCTGAT -3′ |
|  |  | Reverse | 5′- CAGAAGGGTTACGGGCTGAG -3′ |
|  | *ACTB* | Forward | 5′- CATGTACGTTGCTATCCAGGC -3′ |
|  |  | Reverse | 5′- CTCCTTAATGTCACGCACGAT -3′ |
|  | *45s rRNA* | Forward | 5′- CGGGTTATTGCTGACACGC -3 |
|  |  | Reverse | 5′- CAACCTCTCCAGCGACAGG -3 |
